# Liver-inherent immune system: its role in blood-stage malaria

**DOI:** 10.3389/fmicb.2014.00559

**Published:** 2014-11-04

**Authors:** Frank Wunderlich, Saleh Al-Quraishy, Mohamed A. Dkhil

**Affiliations:** ^1^Department of Biology, Heinrich-Heine-University, Düsseldorf, Germany; ^2^Department of Zoology, College of Science, King Saud University, Riyadh, Saudi Arabia; ^3^Department of Zoology and Entomology, Faculty of Science, Helwan University, Cairo, Egypt

**Keywords:** liver, hepatic immune system, tolerance, innate immunity, blood-stage malaria, *Plasmodium*, erythrocytes, “protective” autoimmunity

## Abstract

The liver is well known as that organ which is obligately required for the intrahepatocyte development of the pre-erythrocytic stages of the malaria-causative agent *Plasmodium.* However, largely neglected is the fact that the liver is also a central player of the host defense against the morbidity- and mortality-causing blood stages of the malaria parasites. Indeed, the liver is equipped with a unique immune system that acts locally, however, with systemic impact. Its main “antipodal” functions are to recognize and to generate effective immunoreactivity against pathogens on the one hand, and to generate tolerance to avoid immunoreactivity with “self” and harmless substances as dietary compounds on the other hand. This review provides an introductory survey of the liver-inherent immune system: its pathogen recognition receptors including Toll-like receptors (TLRs) and its major cell constituents with their different facilities to fight and eliminate pathogens. Then, evidence is presented that the liver is also an essential organ to overcome blood-stage malaria. Finally, we discuss effector responses of the liver-inherent immune system directed against blood-stage malaria: activation of TLRs, acute phase response, phagocytic activity, cytokine-mediated pro- and anti-inflammatory responses, generation of “protective” autoimmunity by extrathymic T cells and B-1 cells, and T cell-mediated repair of liver injuries mainly produced by malaria-induced overreactions of the liver-inherent immune system.

## LIVER-INHERENT IMMUNE SYSTEM

The liver is well known for its pivotal role in detoxification of drugs, toxins, and other harmful substances, in protein, lipid, carbohydrate, and vitamin metabolism, in steroidogenesis and bile secretion as well as for its supporting functions of other organs. Less known is the fact that the liver also plays a key immunoregulatory role (Selmi et al., 2007; Crispe, 2009; Nemeth et al., 2009; Parker and Picut, 2012). Indeed, the fetal liver is the primary hematopoietic organ of mammals, and, postnatally, the liver has retained the capability to exert important functions of both innate and adaptive immunity (Parker and Picut, 2012).

The liver-inherent immune system acts locally in the liver, but with systemic implications. Its two major functions are: (i) to sense pathogens, as, e.g., RNA- and DNA-viruses, bacteria, fungi, parasitic protozoans, and to activate efficacious inflammatory and immune responses against these pathogens; (ii) to recognize concomitantly non-pathogenic antigens as in harmless food compounds and to activate delicate mechanisms of immune hyporesponsiveness to generate tolerance (Selmi et al., 2007; Crispe, 2009; Nemeth et al., 2009; Parker and Picut, 2012). Both “antipodal” functions benefit from the dual blood supply of the liver (Nemeth et al., 2009). The hepatic artery delivers approximately 20% oxygenated blood from the circulation, while the portal vein provides approximately 80% venous blood from the gastrointestinal tract. The total blood of the human body is estimated to pass the liver approximately 25 times per hour. During liver passage, the blood percolates through a honeycomb of sinusoids with an approximate diameter of 5–7 μm, by which the velocity of the blood flow is slowed down to 25–250 μm/min (Nemeth et al., 2009). This facilitates the liver cells to recognize blood-delivered pathogens.

Pathogen recognition receptors (PRRs) represent a fundamental part of the liver-inherent immune system, that sense the evolutionary highly conserved pathogen-associated molecular patterns (PAMPs; Broering et al., 2011; Savva and Roger, 2013; Kesar and Odin, 2014). There exists a large reservoir of hepatic PRRs encompassing scavenger receptors (SRs) recognizing their targets by specific glycosylation patterns, modified protein motifs, and lipid moieties (Sorensen et al., 2012), cytoplasmic nucleotide-binding oligomerization domain (NOD)-like receptors recognizing bacterial peptidoglycans, the retinoic acid inducible protein-I (RIG-I) recognizing structural features of viral ssRNA, and, in particular, Toll-like receptors (TLRs; Thompson and Locarnini, 2007). The binding of TLRs by their respective ligands activates innate and ensuing adaptive immune responses. There have been identified 10 functional human TLRs and 12 functional mouse TLRs, with TLRs 1–9 conserved in both humans and mice (Kawai and Akira, 2010).

Toll-like receptors are expressed by the majority of liver cells, though to varying levels (Chen and Sun, 2011). The TLRs 1, 2, 4, 5, and 6 are expressed on cell surfaces, whereas the TLRs 3, 7, 8, and 9 are intracellularly localized in endolysosomal membranes (Kawai and Akira, 2006). The TLRs, except TLR3, signal via the adaptor protein myeloid differentiation factor-88 (MyD88) ultimately activating nuclear factor kappa B (NFκB)-initiated and other pathways.

Effectful responses of the liver-inherent immune system against pathogens are generated by the orchestrated interplay of differently specialized liver-resident cell populations whose main “immune” functions are shortly outlined in the following.

Liver sinusoid endothelial cells (LSECs) form the fenestrated sinusoids-lining endothelium, which separates the sinusoid lumen, without any basement membrane, from the underlying space of Disse and the parenchyma tissue. LSECs represent about 45% of the non-parenchymal cells of the liver (Selmi et al., 2007; Jenne and Kubes, 2013). They express the SRs-A, SR-B, and SR-C as well as different TLRs. In response to ligands for TLRs 1, 2, 4, 6, and 9, LSECs produce tumor necrosis factor α (TNFα), and TLR3 ligands induce interferon-γ (IFNγ), TNFα, and interleukin (IL)-6 (Wu et al., 2010). Moreover, LSECs express major histocompatibility complex (MHC) class I and II molecules, co-stimulatory molecules CD40, CD80, and CD86 and adhesion molecules, as, e.g., intercellular adhesion molecule (ICAM) required for interaction with lymphocytes. Currently, LSECs are envisioned as to represent an intricate platform for antigen presentation (Selmi et al., 2007; Jenne and Kubes, 2013; Nakamoto and Kanai, 2014).

Hepatocytes (HCs) of the liver parenchyma encompass about 70–80% of all liver cells (Nemeth et al., 2009; Jenne and Kubes, 2013). HCs can express TLRs 1–9, though only TLR2 and TLR4 have been reported to be ligand-responsive to date (Liu et al., 2002). HCs induce different innate immune responses (Nemeth et al., 2009; Jenne and Kubes, 2013), but are also able to initiate adaptive immune mechanisms. Indeed, HCs express MHC I molecules, and inflammatory conditions even induce expression of MHC II antigens (Franco et al., 1988), enabling HCs to present antigen to naive T cells (Selmi et al., 2007; Nemeth et al., 2009; Jenne and Kubes, 2013).

Different soluble components produced by HCs majorly contribute to the acute phase response (APR), the first line of innate systemic defense against pathogens. Acute inflammation induces HCs to transiently increase their production of “positive” acute phase proteins (APPs), whereas that of other “negative” APPs are decreased (Steel and Whitehead, 1994; Parker and Picut, 2012). The major positive APPs include serum amyloid A (SAA) and C-reactive protein (CRP) in humans as well as SAA and the CRP-homolog serum amyloid P (SAP) in mice, whose serum concentrations during an APR can increase up to 1000-fold over normal levels (Steel and Whitehead, 1994). Apart of these major APPs, there are numerous other proteins produced in the liver during infection, as, e.g., lipopolysaccharide binding protein, haptoglobin, α2-macroglobulin, different proteinase inhibitors, α1-antitrypsin, and α1-antichymotrypsin (Steel and Whitehead, 1994). Among the negative APPs are the plasminogen activator inhibitor-1 (PAI-1) and the liver isoform of alkaline phosphatase (AP; Steel and Whitehead, 1994). The latter has been used classically as a serum biomarker for hepatic disease states such as hepatitis, steatosis, cirrhosis, drug-induced liver injury, and hepatocellular carcinoma (Pike et al., 2013).

Other soluble substances produced by HCs are complement components, e.g., C3, that opsonizes pathogens for elimination, and C9, that forms the membrane attack complex for final microbe lysis (Parker and Picut, 2012). Moreover, HCs produce a number of circulating growth factors and inflammatory cytokines, amongst which is also IL-6, that in turn induces massive production of APPs by HCs (Parker and Picut, 2012). This pleiotropic cytokine exerts both pro- and anti-inflammatory properties and takes actions through two different signaling mechanisms (Heinrich et al., 2003; Drucker et al., 2010; Scheller et al., 2011). IL-6 “classic signaling” is restricted only to HCs and immune cells, which are activated through their specificity-defining membrane IL-6 receptor α (IL-6Rα) and the recruitment of two chains of the signal-transducing membrane receptor GP130, thus activating the Janus kinase/signal transducers and activators of transcription (JAK/STAT) pathway. However, IL-6 can also communicate with all other cells in the body by a process termed “IL-6 trans-signaling.” Hereby, IL-6 binds to the naturally occurring soluble IL-6Rα, which is derived by shedding of the ectodomain of membrane IL-6Rα and by alternative mRNA splicing in humans, but not mice. The IL-6/IL-6Rα complex can bind to GP130 expressed ubiquituously in surface membranes of all cells. However, the IL-6/sIL-6Rα complex can be inactivated by binding soluble GP130 (sGP130), thus blocking IL-6 trans-signaling (Knupfer and Preiss, 2008).

Kupffer cells (KCs) are located in the sinusoid lumen, adhere to LSECs and represent about 30% of the non-parenchymal liver cells (Parker and Picut, 2012). These liver-resident macrophages constitute about 80–90% of the whole reticuloendothelial system in the human body (Bilzer et al., 2006). The KCs presumably derive from both local hematopoiesis in the liver as well as from bone marrow-derived monocytes that enter the liver and eventually differentiate to liver-resident macrophages (Nemeth et al., 2009; Jenne and Kubes, 2013). KCs are very versatile with a life-span reaching from 1 week until 1 year. They express different surface receptors allowing the detection, binding, and phagoyctosis of pathogens. For instance, KCs express ligand-responsive TLRs 2–4 and TLR9 (Nakamoto and Kanai, 2014), SRs, the complement receptors (CR)1 and CR3 as well as Fc receptors. Even if KCs are not able to internalize and destroy pathogens, they have captured on their surface as, e.g., *Listeria monocytogenes*, they recruit neutrophils from the circulation, which then take over destruction of these pathogens (Gregory et al., 1996). Moreover, KCs are involved in waste disposal, which also includes phagocytosis of activated and apoptotic “self” cells, as, for example, neutrophils, platelets, and T cells. Upon stimulation, KCs produce a series of cytokines among which are TNFα, IL-1β, IL-6, IL-12, and IL-18 (Kopydlowski et al., 1999). Moreover, KCs are capable of antigen presentation. They express MHC I, MHC II and co-stimulatory molecules required for activation of T cells. In particular, KCs can activate natural killer T (NKT) cells patrolling the sinusoids (Jenne and Kubes, 2013).

Hepatic stellate cells (HSCs) or Ito cells reside in the space of Disse, represent about 5–8% of total liver cells, and their dendritic-like protrusions wrap around the sinusoids (Jenne and Kubes, 2013). Apart from vitamin A storing and fibrinogenetic cells, HSCs are able to present lipid antigens to NKT cells, enhance proliferation of NKT cells and may present antigen to CD8^+^ T cells (Nemeth et al., 2009; Jenne and Kubes, 2013). Upon activation, human HSCs express TLR4, along with CD14, and the mouse HSCs TLRs 2 and 4 express vascular cell adhesion molecule 1 (VCAM-1), transforming growth factor β1 (TGF-β1) and monocyte chemoattractant protein-1 (MCP-1; Selmi et al., 2007; Jenne and Kubes, 2013).

Dendritic cells (DCs) are concentrated around the portal triad and around the central veins of the liver. There exist at least five DC-types of with different “immature” phenotypes: lymphoid DCs (CD8a^+^ B220^–^ CD11b^+^), myeloid DCs (CD8^–^ B220^–^ CD11b^+^), plasmacytoid DCs (CD8a^–^ B220^+^), mixed lymphoid/myeloid DCs (B220^–^ CD11b^–^), and natural killer (NK) DCs (B220^–^ CD11c_int_ CD69^+^ 2B4^+^ DX5^+^; Selmi et al., 2007; Nemeth et al., 2009; Jenne and Kubes, 2013). Hepatic DCs differ from their counterparts in any classical lymphoid organ not only by their immature phenotype, but also by less immunogenicity, higher productions of cytokines, and higher endocytotic activity (Hsu et al., 2007). They produce TNFα and IL-6 in response to diverse ligand-activated TLRs. The ability of DCs to activate T cells can be impaired by LSECs, which may be important for induction and maintenance of immune tolerance. The major function ascribed to hepatic DCs is to control the balance between oral and portal autoimmune diseases (Selmi et al., 2007; Nemeth et al., 2009).

Liver-resident lymphocytes, i.e., NK cells, NKT cells, T cells, and B cells, account for about 25% of the non-parenchymal cells of the liver. They differ from their corresponding cell populations in other tissues in several aspects (Selmi et al., 2007; Crispe, 2009; Jenne and Kubes, 2013).

Natural killer cells are large granular lymphocytes (LGLs), represent about 35–50% of all liver-resident lymphocytes and comprise a very heterogenous pattern (Nemeth et al., 2009). NK cells in the liver are also more frequent than in any other tissues. For instance, these innate lymphocytes are enriched more than threefold in comparison to those in blood (Crispe, 2009). They possess diverse PRRs which do not only recognize PAMPs, but also damage-associated molecular patterns (DAMPs). NK cells express ligand-responsive TLRs 1–4 and TLRs 6–9 (Sawaki et al., 2007). Upon activation and in very close contact with target cells, which may be opsonized by antibodies, NK cells release perforin and various granzymes from their intracellular vesicle stores by exocytosis, which kills foreign microbes (Lodoen and Lanier, 2006) and also malignant cells (Herbermann et al., 1975; Notas et al., 2009). In addition, activated NK cells secrete numerous cytokines, as, e.g., IFNγ, and are therefore considered as to shape subsequent immune responses including modulation of MHC expression by HCs and HSCs (Crispe, 2009).

Natural killer T cells are an abundant population amongst liver-resident lymphocytes (Crispe, 2009; Nemeth et al., 2009; Jenne and Kubes, 2013). Besides characteristic NK markers, NKT cells also express a limited repertoire of T cell receptors (TCRs). They are mainly CD4^+^, are restricted by CD1d molecules, and display a LGL-morphology. There exist two populations, i.e., NK1.1^+^ TCR^int^ (NKT cells) and NK1.1^–^ TCR^int^ (Abo et al., 2012). Invariant NKT (iNKT) cells express the invariant TCRα (Vα14-Jα18) in mice and Vα24-Jα18 in humans and respond to various lipids in context with the non-classical MHC I molecule CD1d (Bendelac et al., 2007). The CD1-dependent iNKT make up to 30% of total hepatic lymphocytes, whereas their content in blood is only 0.5%. Another group of NKT cells are the CD1d-independent NK1.1^+^ T cells. Hepatic NKT cells are characterized by peculiar features: (i) They apparently are the only cell populations among the liver-resident lymphocytes, which actively patrol the hepatic vasculature for detecting pathogens. (ii) They can also detect insults in further distant tissues and then act as modulators of the global immune status. (iii) They might play a role in liver autoimmune diseases upon tolerance breakdown (Nemeth et al., 2009; Abo et al., 2012).

T cells contribute about 25% to the liver-resident lymphocytes (Selmi et al., 2007; Crispe, 2009; Nemeth et al., 2009). Most of them are extrathymic T cells developing outside the thymus and express only intermediate amounts of TCR (Seki et al., 1991; Abo et al., 2012). CD8^+^ T cells are more abundant than CD4^+^ T cells in the liver (Selmi et al., 2007). Moreover, double negative CD4^–^ CD8^–^ T cells occur in the liver and many of them express _γδ_ TCR instead of the conventional _αβ_ TCR (Nemeth et al., 2009). Most hepatic T cells have an activated phenotype; for instance, CD25 and CD69 are expressed on CD8^+^ T cells. The T cells in the liver differ in general from those in blood, lymph nodes, and spleen by a lower ratio of CD4/CD8, higher percentage of double positive CD3^+^ CD4^+^ CD8^+^ and double negative CD3^+^ CD4^–^ CD8^–^, as well as higher abundance of _γδ_TCR cells. The latter represent about 15–25% of hepatic T cells, but only 3% of blood T cells (Nemeth et al., 2009). T cells can be activated through their TLRs 2, 3, and 9 (Gelmann et al., 2004; Komai-Koma et al., 2004). Importantly, the liver also contains several subsets of regulatory T (T_reg_) cells, and specific T_reg_ cells are assumed to play an important role in induction and maintenance of tolerance (Selmi et al., 2007; Crispe, 2009).

B cells represent less than 10% of the liver-resident lymphocytes in the human liver. In mice, the fetal and adult liver contains B220^low^ B-1 cells, being either CD5^+^ or CD5^–^ (Tachikawa et al., 2008; Baumgarth, 2011; Abo et al., 2012), with CD5 as a negative regulator of B cell receptor signaling (Lankester et al., 1994). Incidentally, B-1 cell development is negatively regulated by the nuclear pregnane X receptor in the liver (Casey and Blumberg, 2012). The majority of intrahepatic B cells resembles conventional B220^high^ B-2 lymphocytes. B-2 cells are IgM^low^ IgD^high^ CD23^+^, while B-1 cells are IgM^high^ IgD^low^ CD3^–^ (Tachikawa et al., 2008). B-1 and B-2 cells produce low affinity autoreactive IgM antibodies, also termed natural antibodies (Baumgarth et al., 2000). Most natural antibodies are polyreactive with numerous “self” and “non-self” antigens and are involved in efficient control of pathogens from circulation (Baumgarth et al., 2000), in the clearance of senescent erythrocytes (Lutz et al., 1990), in removal of apoptotic bodies (Duan and Morel, 2006), and in autoimmune diseases (Ochsenbein and Zinkernagel, 2000).

## THE LIVER: EFFECTOR ORGAN AGAINST BLOOD-STAGE MALARIA

The malaria-causative agents are parasitic protozoans of the genus *Plasmodium.* These are transmitted by *Anopheles* and reach via the bloodstream the liver, in which they invade HCs and undergo asexual multiplication (Frevert and Nacer, 2013). This pre-erythrocytic development of the parasites in the liver proceeds clinically asymptomatic, it is not preventable by the liver-inherent immune system, though it may possibly benefit, at least transiently, from the tolerogenic properties of the liver (Frevert, 2004). Then, merozoites are released from HCs, which penetrate erythrocytes and the ensuing asexual multiplication in erythrocytes is associated with morbidity and mortality of malaria.

Blood-stage malaria afflicts about 225 million people and kills about 781,000 people, mainly African children, worldwide per annum (Garcia, 2010; WHO, 2010). The majority of the malaria-afflicted people can obviously cope with the infections. Repeated blood-stage infections in malaria endemic areas obviously lead, though slowly, to acquisition of natural immunity against the blood-stage malaria. This natural immunity is not solid: it reduces disease symptoms, but it does not prevent re-infections with elevated blood parasitemias during malaria season. It is specific for species and strain of *Plasmodium*, and it is short-lived (Cohen and Lambert, 1982). It appears as if the slow acquisition of natural immunity may be associated with increasing tolerance generated by the liver against malarial blood-stages, though experimental evidence for this view is lacking.

The spleen is currently considered as to be the central effector site of host immunity against blood-stage malaria (Chotivanich et al., 2002; Engwerda et al., 2005; Del Portillo et al., 2012). The spleen is equipped with mechanisms to eliminate senescent and aberrant red blood cells (RBCs) including parasitized RBCs (pRBCs). However, previous studies with the experimental malaria *P. yoelii* have already revealed that mice, after vaccination and splenectomy, are still able to clear blood-stage infections, albeit at delayed recovery (Playfair et al., 1979; Dockrell et al., 1980). Also, splenic uptake of injected labeled pRBCs during infections with *P. yoelii* decreased with progressing course of infection. Concomitantly, however, the liver has increased its uptake of pRBCs.

Similarly, it has been found in *P. chabaudi*-infected mice that, during the crisis phase of infection, which is characterized by a dramatic decline of peripheral parasitemia from about 35–55% at peak parasitemia to about 1–2% within 3–4 days, the spleen, though dramatically enlarged, becomes “closed” for entry of particulate material. Indeed, the spleen excludes uptake of injected labeled pRBCs or inert polystyrol beads, but not that of fluorescently labeled bovine serum albumin (Krücken et al., 2005, 2009). Concomitantly, however, the liver has increased its trapping capacity of particles (Krücken et al., 2005, 2009). Moreover, the importance of the liver against blood-stage malaria is particularly demonstrated in lymphotoxin β receptor (LTβR)-deficient mice which have severe defects of the spleen and have also lost all other secondary lymphoid organs (Fütterer et al., 1998). Nevertheless, these LTβR-deficient mice are even more resistant to blood-stage malaria of *P. chabaudi* than the corresponding control mice (Wunderlich et al., 2005). These findings support the view that the liver with its immanent immune system is also a major—if not essential—player of the host defense against blood-stage malaria, though this aspect has been largely neglected by research to date.

## LIVER EFFECTORS TOWARD BLOOD-STAGE MALARIA

Evidence is increasing that the liver-resident immune system is obviously able to respond to blood-stage malaria with the development of efficient anti-malaria effector mechanisms.

Toll-like receptors are activated in the liver by blood-stage malaria. Indeed, experimental malaria with *P. chabaudi* induces significant increases in mRNA expression of *Tlr 1*, *2*, *4*, *6*, *7*, and *8*, varying between 4- and 21-fold in the liver of C57BL/6 mice (Al-Quraishy et al., 2014). By contrast, the expression of *Tlr3*, *Tlr5*, and *Tlr9* does not respond to malaria infection at all. It is conspicuous that the mRNA levels of both the surface-localized TLR1, TLR2, and TLR6, as well as the intracellular TLR7 and TLR8 are increased.

TLR1 and TLR6 are currently regarded as auxiliary receptors, which both form heterodimers with TLR2 in the plasma membrane (Kawai and Akira, 2010; Oliveira-Nascimento et al., 2012). These heterodimers of both TLR2/1 and TLR2/6 recognize a broad spectrum of different PAMPs, in particular multiple diacyl lipopeptides of various infectious agents such as viruses, bacteria, fungi, and parasites (Oliveira-Nascimento et al., 2012; De Almeda et al., 2013; Misch et al., 2013; Zhang et al., 2013). PAMPs recognition then activates a cascade of downstream reactions eventually leading to innate and adaptive immune responses directed against pathogens and, also, to protection from those adverse processes induced by host responses to infections (Kawai and Akira, 2010; Oliveira-Nascimento et al., 2012). In human malaria with *P. falciparum*, the TLR2/1 and TLR2/6 complexes recognize specific components of the glycosylphosphatidylinositols (GPIs), that are regarded as major factors contributing to malaria pathogenesis (Krishnegowda et al., 2005). Thus, TLR2/1 recognizes intact 3-acyl-GPIs and TLR2/6 the sn-2-lyso-GPI components (Krishnegowda et al., 2005). Remarkably, expression of *Tlr6* and *Tlr1*, but not that of *Tlr2*, *Tlr7*, and *Tlr8*, are under epigenetic control, since blood-stage infections of *P. chabaudi* induce alterations of the DNA methylation status of the promoters of *Tlr1* and *Tlr6* genes (Al-Quraishy et al., 2013). This malaria-induced epigenetic fine-tuning of *Tlr6* and *Tlr1* in the liver may be an important initial step of the host response against *Plasmodium* blood stages. Since DNA methylation is stable (Bird, 2002), it may be further suspected that epigenetic fine tuning of *Tlr6* and *Tlr1* may contribute to memory against homolog re-infections.

In contrast to *Tlr1* and *Tlr6*, the DNA methylation status of the *Tlr8* gene promoter is not affected by *P. chabaudi* infections at all. The function of TLR8, intracellularly localized in endosomal membranes, are not yet well understood (Cervantes et al., 2012; Kesar and Odin, 2014). TLR8 has been shown to sense single-stranded RNA of viral origin and bacterial RNA as well as to induce NFκB-dependent cytokines and type I IFNs. However, increasing information suggests that TLR8, possibly in cross-talk with TLR7 (Wang et al., 2006), is involved in the generation of autoimmunity (Krieg and Vollmer, 2007; DeMaria et al., 2010). Remarkably, dysregulations of *Tlr8* and *Tlr6* occur, when *P. chabaudi* infections are induced by testosterone to take a lethal outcome in female mice (Al-Quraishy et al., 2014).

### ACUTE PHASE RESPONSE

An APR is experimentally inducible by blood-stage malaria, without any preceding pre-erythrocytic development of parasites in the liver, in humans and model systems. When in human volunteers an APR is induced by injecting *P. falciparum*-infected RBCs, serum levels of α1-acid glycoprotein (AGP) peak within 1 week (Klainer et al., 1968). AGP inhibits *in vitro* growth of *P. falciparum* by more than 80% at a concentration of 2.5–3.0 mg/ml, which resembles those levels reached during a typical APR in humans (Friedman, 1983). Other APPs such as α1-antitrypsin, transferrin or α2-macroglobulin have been reported to be not as effective against *P. falciparum* *in vitro* (Friedman, 1983). Patients suffering from malaria reveal elevated serum CRP which binds to the patients’ RBCs. These in turn loose their normal discoid-shape, increase membrane fluidity and hydrophobicity and decrease their effective complement-regulatory proteins (Ansar et al., 2009). In mice, blood-stage infections of both self-healing *P. chabaudi* and *P. vinckei* as well as lethal *P. berghei* induce an APR characterized by increased serum levels of the major APP SAP (Balmer et al., 2000). In accordance, the mRNA levels of *Saa 1–3* in the liver increased by 65- to 80-fold at peak parasitemia of *P. chabaudi* infections (Krücken et al., 2005). By contrast, a reduced APR in association with a significant extension of primary parasitemia is induced in IL-6-deficient mice in response to blood-stage infections. This suggests that the APR induced by blood-stage infections contributes to an antiparasitic and/or immunomodulatory immune response (Balmer et al., 2000).

It is remarkable, however, that negative APPs, as, e.g., haptoglobin, PAI-1, and AP with potentially efficient anti-malaria activity, are decreased during the APR against blood-stage malaria. For instance, haptoglobin is malaria-protective, since it is toxic to *P. falciparum*-infected RBCs. Its main function is to bind hemoglobin released from erythrocytes and to support its elimination, thereby also preventing oxidative stress in the blood (Imrie et al., 2004). Hypohaptoglobinemia has been introduced as a biochemical and epidemiological marker of *P. falciparum* malaria (Mohapatra et al., 1999). In experimental *P. chabaudi* malaria, the *Pai-1* gene is downregulated in the liver. Since *Pai-1*-deficient mice cannot control parasitemia as good as wild type mice, PAI-1 has been suggested to be required for parasitemia control (Krücken et al., 2005). AP is also reduced at peak parasitemia during self-healing *P. chabaudi* infections (Wunderlich et al., 2005). AP exerts anti-inflammatory effects, as it is capable of dephosphorylating potentially deleterious molecules and of reducing inflammation and coagulopathy systemically (Pike et al., 2013). Obviously, the blood stages of the malaria parasites benefit from the down-regulation of negative APPs during an APR.

### INFLAMMATORY RESPONSE

Course and outcome of both human and experimental blood-stage malaria critically depend on a finely tuned balance between both pro-inflammatory cytokines, as, e.g., IL-1β, TNFα, IFNγ, IL-6, and IL-12, and anti-inflammatory cytokines such as IL-4 and IL-10 (Bakir et al., 2011; Perkins et al., 2011).

In different experimental models, the liver has been shown to produce different cytokines in response to primary blood-stage malaria. However, there are differences between lethal and self-healing infections. In lethal *P. chabaudi* malaria, there occurs biphasic, two-wavy increase in serum IL-1β, TNFα, and IL-6 with the first wave peaking on day 1 *p.i.* and the second wave peaking at maximal parasitemia on day 8 *p.i.* (Wunderlich et al., 2012). In parallel, the liver biphasically produces mRNAs coding for IL-1β, TNFα, and IL-6 (Krücken et al., 2009). By contrast, the hepatic production of IFNγ mRNA reveals a lower and more uniform increase during precrisis. This pattern of mRNA production is totally reverted after protective vaccination of mice. Then, the liver produces a pronounced biphasic increase of IFN mRNA at higher levels, while the mRNA levels of IL-1β, TNFα, and IL-6 are lower and the biphasic production pattern has largely disappeared (Krücken et al., 2009). This indicates that protection against blood-stage malaria is associated with low production of IL-1β, TNFα, IL-6 and a high biphasic production IFNγ by the liver. Incidentally, a two-wavy production pattern, as well as its disappearance after protective vaccination, can be also found for the mRNA production of inducible nitric oxide synthase (iNOS), arginase, and different nuclear receptors including pregnane X receptor in the liver (Krücken et al., 2009).

In mice infected with a non-lethal strain of *P. yoelii*, the liver has been also shown to produce mRNAs of both pro-inflammatory cytokines, i.e., IFNγ, IL-12 p40, IL-6, and TNFα, and anti-inflammatory cytokines such as IL-4 and IL-10 (Bakir et al., 2011). Both pro- and anti-inflammatory cytokines are highly expressed during precrisis of blood-stage infections. It has been suggested that the pro-inflammatory cytokines induce the immune system to take action, while the anti-inflammatory cytokines counteract the high levels of inflammatory cytokines (Bakir et al., 2011).

The liver-produced cytokines are not only important for the local response in the liver, but also have presumably an impact for the systemic response to blood-stage malaria. For instance, a rather complex and dynamic interplay has been evidenced for IL-6. This pleiotropic cytokine is produced not only by HCs, but also by KCs and other liver cells. The severity of blood-stage malaria correlates with circulating IL-6 levels. Increased levels have been described in patients suffering from malaria caused by *P. falciparum* and *P. vivax* (Kern et al., 1989; Jason et al., 2001; Lyke et al., 2004; Robinson et al., 2009), often associated with polyclonal B cell activation (Donati et al., 2004). Conversely, decreasing IL-6 levels are reported to be associated with decreasing parasitemia (Sarthou et al., 1997) and hyperpyrexia (Seoh et al., 2003), as well as after anti-malarial treatment (Hugosson et al., 2006). In experimental murine malaria, it has been shown that IL-6 trans-signaling, rather than classic IL-6 signaling, contributes to malaria-induced lethality (Wunderlich et al., 2012). Indeed, approximately 50% of IL-6Rα-deficient mice survive an otherwise lethal *P. chabaudi* blood-stage malaria. However, the lethal outcome is restored, when IL-6 trans-signaling is induced by injecting mouse recombinant sIL-6Ra in the IL-6Rα-deficient mice. By contrast, inhibition of IL-6 trans-signaling through injecting recombinant sGP130Fc protein in wild type mice results in 40% survival (Wunderlich et al., 2012).

### PHAGOCYTIC ACTIVITY

Intraerythrocytic stages of *Plasmodium* produce and store hemozoin in their phagosomes to detoxify the lytic ferriprotoporphyrin IX released during digestion of host hemoglobin by parasites (Olivier et al., 2014). Since ferriprotoporphyrin IX is also lytic for host cells including human RBCs (Orjih et al., 1988), the hemozoin is therefore cleared from the circulation as potentially dangerous waste material by phagocytosis of KCs to protect host cells.

On the other hand, KCs are also known to be endowed with the capacity to phagocytose and to remove senescent and aberrant erythrocytes including pRBCs from circulation (Otogawa et al., 2007; Lee et al., 2011) mediated by specific SRs (Terpstra and van Berkel, 2000). It has been therefore suspected that at least part of the intracellularly accumulated hemozoin in KCs is derived from internalized and decayed pRBCs. This view is supported by several findings. In rats with a high load of *P. berghei* blood-stage infections, KCs exhibit a markedly increased phagocytic activity, presumably due to an upregulation of scavenger activity for erythrophagocytosis (Nobes et al., 2002). Moreover, it has been shown in *P. berghei*-infected rats that the number of KCs is significantly increased and the phagocytic activity of KCs on a cell-by-cell basis is enhanced (Murthi et al., 2006). Furthermore, at peak parasitemia of *P. chabaudi*-infected mice, KCs are characterized by hyperplasia, and the massively enlarged KCs take on a “foamy” appearance detectable by light microscopy (Delic et al., 2010). These KCs contain, besides hemozoin, also regularly pRBCs inside the area of sectioned KCs thus supporting the view that KCs are indeed able to erythrophagocytose pRBCs and to destroy them in their phagosomes *in vivo.*

Several studies in non-KC macrophages have shown that ingestion of hemozoin by macrophages severely impairs macrophage activities upon diverse stimulations. For instance, hemozoin dramatically reduces the production of IL-6, but increases production of TNFα (Prada et al., 1995) and inhibits activities of NADPH-oxidase activity (Schwarzer and Arese, 1996) and protein kinase C (Schwarzer et al., 1993). Moreover, the production of reactive oxygen species (ROS) and reactive nitrogen species (RNS) is inhibited (Schwarzer et al., 1993; Prada et al., 1996) and the expression of MHC II molecules, CD54 and CD11c is impaired (Schwarzer et al., 1998). However, hemozoin does not specifically affect expression of MHC I molecules, CD16 (low affinity Fc receptor for aggregated IgG), CD64 (high affinity receptor for IgG), CD11b (receptor for complement receptor iC3b), CD35 (receptor for complement components C3b and C4b), and CD36 (non-class-A SR; Schwarzer et al., 1998; Olivier et al., 2014). More recent data indicate that hemozoin can induce the production of IL-1β through the NOD-like containing pyrin 3 domain (NLRP3) inflammasome complex (Olivier et al., 2014). These data suggest that also *in vivo* activities of KCs may be negatively affected by ingested hemozoin. Indirect evidence for this view is provided by a magnetometric and electron microscopal study showing that ingestion and metabolism of *P. chabaudi*-infected RBCs by KCs *in vivo* decrease intracellular organelle motion in these KCs, which probably compromises host defense reactions (Bellows et al., 2011).

### “PROTECTIVE” AUTOIMMUNITY

In humans, acquisition of natural immunity against blood-stage malaria is associated with the emergence of autoimmunity (Butcher, 2008). A number of studies with murine blood-stage malaria have substantiated the view that autoimmunity arises during blood-stage malaria and that the liver-inherent immune system is critically involved in the generation of this “anti-malaria” autoimmunity. This is presumably not mediated by conventional T and B cells, but rather by special populations of hepatic T and B cells of innate immunity (Abo et al., 2012). For instance, *P. yoelii* infections have been reported to induce severe thymic atrophy in mice, which ultimately arrests production of conventional T cells (Abo et al., 2012). Concomitantly, however, extrathymic IL-2Rb^+^ CD3^int^ T cells and its subset NK1.1^–^ CD3^int^ expand in the liver. Moreover, NK1.1^–^ TCR^int^ cells are present in the liver of athymic nude mice, which harbor neither conventional T cells nor NKT cells of thymic origin. When lymphocytes are isolated from the liver of athymic mice recovered from blood-stage malaria of non-lethal *P. yoelii*, these “immune” lymphocytes can confer protection from malaria to both irradiated euthymic and athymic mice (Mannoor et al., 2002). Furthermore, autoreactive extrathymic T cells have been described in the liver of malaria-infected mice, which react with both HCs and RBCs of malaria-infected mice (Abo et al., 2012).

In parallel with the expansion of extrathymic autoreactive T cells, the number of B220^low^ B-1 cells is also increased in the liver and is highest around peak parasitemia in mice infected with non-lethal *P. yoelii* (Kanda et al., 2010; Abo et al., 2012). These B-1 cells produce autoantibodies against nucleus, ss- and dsDNA (Kanda et al., 2014). In accordance, serum levels of both IgM- and IgG-type autoantibodies against denatured DNA increased during non-lethal *P. yoelii* infections in mice, both peaking around peak parasitemia (Bakir et al., 2011). These immune responses resemble those found in autoimmune diseases (Bakir et al., 2011). *In vivo* depletion of autoreactive B-1 cells and subsequent infection with normally self-healing *P. yoelii* result in death of 56% of mice (Mannoor et al., 2013). By contrast, adoptive transfer of such auto-antibody secreting B cells prior to infecting with *P. yoelii* leads to later appearance and inhibition of parasitemia (Mannoor et al., 2013). Auto-antibodies secreting B220^low^ B-1 cells have been suggested to target “abnormal self” cells, as, e.g., HCs and RBCs, in malaria-infected mice, and such autoantibody-labeled cells can be further processed and eliminated by macrophages and KCs (Kanda et al., 2014).

Previous authors have already suggested that autoimmunity plays a key regulatory role in protection from malaria (Jayawardena et al., 1979; Jarra, 1980). In accordance, it has been recently found that autoantibodies and sera from patients suffering from diverse autoimmune conditions, but not from malaria, inhibit the *in vitro* growth of *P. falciparum* in human RBCs (Bhatnagar et al., 2011; Brahimi et al., 2011). Especially that type of autoimmunity is apparently required for protection against blood-stage malaria (Daniel-Ribeiro, 2000), that is presumably directed against autoantigens and parasite-induced neo-autoantigens on the surface of *Plasmodium*-infected RBCs (Wunderlich et al., 1988b,c; Fontaine et al., 2012). It is therefore not surprising that surface membranes of *Plasmodium*-infected RBCs can be used for protective vaccination of malaria-susceptible mice, which raises their survival from 0 to more than 80% and decreases peak parasitemia by about 30% (Wunderlich et al., 1988a; Krücken et al., 2009).

Collectively, the currently available information supports the view that the generation of autoimmunity and/or the enhancement of already existing low level “natural” autoimmunity in the liver may be an integrative—if not the essential—part of protective immunity against blood-stage malaria (Abo et al., 2012). Malaria-activated TLR8, possibly in cross-talk with TLR7, has been speculated to be involved in the generation and/or surveillance of this “protective” autoimmunity (Al-Quraishy et al., 2014).

### LIVER INJURIES AND REPAIR

Blood-stage malaria causes severe injuries of the liver. Humans infected with *P. falciparum* or *P. vivax* have been described to suffer from hepatic dysfunctions and malarial hepatitis with jaundice and raised liver enzyme levels in the blood (Ananad et al., 1992; Kochar et al., 2003; Nautiyal et al., 2005). Liver injuries have been also found *postmortem* in fatal human malaria, as, e.g., KC hyperplasia, hemozoin accumulation within KCs, liver-necrosis, portal inflammation, steatosis, and cholestasis (Rupani and Amarapurkar, 2009). Similar histopathological injuries have been also observed in the liver of mice primarily infected with lethal *P. chabaudi* at peak parasitemia, besides increased serum levels of hepatic markers such as aspartate aminotransferase (AST), alanine aminotransferase (ALT), bile acids, and bilirubin (Krücken et al., 2005; Delic et al., 2010). However, similar changes, though not as pronounced, also occur in the liver of mice when *P. chabaudi* infections take a self-healing course (Krücken et al., 2005; Delic et al., 2010).

Liver injuries are presumably not directly induced by the parasites, but largely indirectly as infection-induced overreactions of the liver-inherent immune system, as, e.g., increased oxidative stress induced by ROS/RNS produced during phagocytic activity of KCs, increased production of proinflammatory cytokines, increased release of perforin, granzymes, and lysosomal enzymes by LGL, autoreactive cytotoxic T cells, and “bystander killing” of liver cells due to cell-mediated cytotoxicity. For instance, hepatic CD1d-independent DX5^+^ T cells have been shown to cause “bystander killing” in the liver of *P. berghei*-infected mice (Adachi et al., 2004). Hepatotoxicity can be also induced by hepatic CD1d-restricted NKT cells activated during blood-stage malaria through IL-12 secreted from KCs (Gonzalez-Aseguinolaza et al., 2000). Damages of liver cells have been suggested to be surveilled by DAMPs-activated TLRs (Adachi et al., 2001).

Even an apparent “non-immune” mechanism generating liver injury is very likely immune-mediated, as, for example, the almost doubling of the ammonia content in the blood plasma found at peak parasitemia during lethal *P. chabaudi* malaria (Delic et al., 2010). The increased systemic ammonia level signalizes severe local hepatic dysfunctions. Indeed, the liver metabolism is impaired, in particular the detoxifying capacity of the liver, evidenced as massive down-regulations of genes encoding enzymes involved in phase I–III metabolism (Krücken et al., 2005; Delic et al., 2010). However, similar changes in liver metabolism can be experimentally induced in mice only by injections of TNFα and IL-1β without any malaria infection (Kim et al., 2004, 2007). Increases in systemic ammonia levels presumably also cause dysfunctions of other organs. For instance, the brain is particularly sensitive to ammonia intoxication, which ultimately leads to hepatoencephalopathy (Butterworth, 2002; Häussinger and Schliess, 2008), that has been indeed reported to occur as a severe complication in *P. falciparum*-infected patients (Kochar et al., 2003; Whitten et al., 2011).

However, the liver also possess the capability for fast regeneration of destructed liver tissue. This capability also involves T cell-mediated repair mechanisms of the liver-inherent immune system. For instance, *P. chabaudi* blood-stage infections in mice have been described to induce an increase of liver-resident IL-17- and IL-22-producing CD8^+^ T cells (Mastelic et al., 2012). In contrast to IL-17 producing T cells, the IL-22 producing T cells very likely protect from the potentially lethal effects of liver damages during primary *P. chabaudi* infections. Further work is required to disentangle those host mechanisms in the liver, which mediate repair and healing of liver tissue injured during blood-stage malaria, and those, which contribute to liver dysfunctions and ultimately to multiple organ failure as the reason for malaria mortality (Delic et al., 2010).

## CONCLUDING REMARKS

The importance of the liver-inherent immune system as part of the host defense against blood-stage malaria is increasingly recognized, though still too hesitatingly. The liver-inherent immune system is able to develop several different effector responses against malarial blood stages. Development and efficacy of these blood-stage effectors may be modulated by those immune responses which are previously elicited against the pre-erythrocytic liver stages of the malaria parasites (Klotz and Frevert, 2008) and, conversely, the latter may be even boosted by the blood-stage effectors (Ocana-Morgner et al., 2003; Lau et al., 2014).

Overreactions of the effector responses directed against blood-stage malaria presumably lead to severe inflammatory liver injuries having systemic impact. Such overreactions are puzzling since the liver-inherent immune system is also known for its capability to generate tolerance, a state of immune hyporesponsiveness. The “tolerogenic” milieu of the liver may be considered to be beneficial for blood-stage parasites of different *Plasmodium* species including *P. falciparum* and *P. chabaudi* to escape anti-malaria effectors by intrahepatic sequestration (Medeiros et al., 2013; Brugat et al., 2014). Though intrahepatic sequestration mediated by CD36 has been reported to be fundamentally important for parasite growth *in vivo* (Fonager et al., 2012), parasite sequestration also occurs in other “non-tolerogenic” organs.

It is also puzzling how a tolerogenic hyporesponsive milieu as in the liver allows the generation of specific autoimmunity at all, that is presumably even an integrative—if not the essential—part of protective immunity against blood-stage malaria. This autoimmunity is mediated by autoreactive extrathymic T cells and autoantibody-producing B-1 cells. One target of autoimmunity is the RBC, the host cell of the malaria blood stages. Remarkably, surface membranes of *Plasmodium*-infected RBCs, but not those of non-infected RBCs, can be used for protective vaccination of mice (Wunderlich et al., 1988a; Krücken et al., 2009). This raises survival of malaria-susceptible mice from 0 to more than 80%, decreases parasitemia and concomitantly attenuates overreactions of hepatic inflammation induced by blood-stage malaria of *P. chabaudi.* This vaccination model appears rather convenient to trace those immune and possible autoimmune mechanisms, which have to be activated and/or suppressed in the liver to generate efficient protection against blood-stage malaria. Such knowledge in turn will advance our understanding of how these mechanisms can be utilized for the design of an effective human anti-malaria vaccine.

### Conflict of Interest Statement

The authors declare that the research was conducted in the absence of any commercial or financial relationships that could be construed as a potential conflict of interest.
